# Decadal-scale variability and warming affect spring timing and forest growth across the western Great Lakes region

**DOI:** 10.1007/s00484-023-02616-y

**Published:** 2024-01-18

**Authors:** Mara Y. McPartland

**Affiliations:** 1 Alfred-Wegener-Institut Helmholtz-Zentrum für Polar- und Meeresforschung, Potsdam, Germany; 2 Department of Geography, Environment & Society, University of Minnesota, Minneapolis, MN USA

**Keywords:** Climate change, Variability, Spring, Forests, Tree-rings, NDVI

## Abstract

The Great Lakes region of North America has warmed by 1–2 °C on average since pre-industrial times, with the most pronounced changes observable during winter and spring. Interannual variability in temperatures remains high, however, due to the influence of ocean-atmosphere circulation patterns that modulate the warming trend across years. Variations in spring temperatures determine growing season length and plant phenology, with implications for whole ecosystem function. Studying how both internal climate variability and the “secular” warming trend interact to produce trends in temperature is necessary to estimate potential ecological responses to future warming scenarios. This study examines how external anthropogenic forcing and decadal-scale variability influence spring temperatures across the western Great Lakes region and estimates the sensitivity of regional forests to temperature using long-term growth records from tree-rings and satellite data. Using a modeling approach designed to test for regime shifts in dynamic time series, this work shows that mid-continent spring climatology was strongly influenced by the 1976/1977 phase change in North Pacific atmospheric circulation, and that regional forests show a strengthening response to spring temperatures during the last half-century.

**Supplementary Information:**

The online version contains supplementary material available at 10.1007/s00484-023-02616-y.

## Introduction

### Great Lakes climatology during the seasonal transition from winter to spring

Across the Laurentian Great Lakes region, the most pronounced changes in temperature in recent decades compared with the pre-industrial period have occurred during the winter and spring (Moss and Blumenfeld 2017; Wuebbles et al. 2017). Across the northern plains and Great Lakes, the frost-free season has lengthened by over 10 days on average since the beginning of the instrumental period, concurrent with the Northern Hemisphere warming trend (Kunkel et al. 2004; Yu et al. 2014; Wuebbles et al. 2017). However, the “secular” warming trend is modulated by substantial internal, or natural, climate variability on time scales ranging from weeks to decades that may either dampen regional warming or amplify it to produce more extreme values (Ghil and Vautard 1991; Simolo and Corti 2022). Determining how interannual-to-decadal scale variability modifies or exacerbates the warming trend is an important step in characterizing the full range of possible climate conditions for any given region.

Despite its continental location, central North America is influenced by atmospheric teleconnections from remote centers of ocean-atmosphere variability. The Northern Annular mode (NAM) and Pacific North-America (PNA) pattern, both major modes of decadal-scale variability centered over the Arctic and North Pacific oceans, respectively, influence the strength and direction of Rossby waves reaching the continental interior (Trenberth and Hurrell 1994; Thompson and Wallace 1998, 2001; McAfee and Russell 2008; Stendel et al. 2021). The phases of the NAM determine the strength and direction of zonal winds and winter storm tracks across Central and Eastern North America, affecting lake levels and ice-out dates on the Great Lakes (Assel et al. 2003; Ghanbari and Bravo 2008; Saber et al. 2023). The PNA is the tropospheric expression of variation in the Aleutian low-pressure system which varies in conjunction with sea surface temperature (SST) anomalies across the Pacific basin (Wallace and Gutzler 1981; Trenberth and Hurrell 1994; Trenberth et al. 1998; Deser et al. 2004; Yu and Zwiers 2007). Evidence for the influence of Pacific atmospheric circulation on the Great Lakes is mixed: Yu et al. (2014) found that PNA variability explained 30% of the variance in the date of the region’s last spring frost. Ault et al. (2015) also examined links between spring timing and atmospheric modes of variability in the USA but concluded that the secular warming trend was likely responsible for the observed changes in spring, but that internal variability did not play a significant role. Reducing uncertainty around how climate dynamics influence seasonal temperatures on local-to-regional scales is necessary to constrain future estimates of regional climate change (Deser et al. 2012; Yu et al. 2020; Maher et al. 2020).

### Linking spring temperatures to forest phenology and function

Warming in spring affects plant life cycles, (i.e., phenology), by facilitating earlier leaf-out dates and extending the number of possible days of photosynthetic activity (Easterling 2002, Schwartz et al. 2006, 2012; Schwartz and Reiter 2000: Cleland et al. 2007). Changes in spring phenology affect whole ecosystems by altering plant-animal interactions and rates of carbon sequestration and storage (Piao et al. 2007; Polgar and Primack 2011; Hänninen 2016). In temperate mesic forests in the Northeast, the longer growing season has been associated with an increase in annual net primary productivity during the past half-century, although the effects on whole ecosystem carbon accumulation remain unclear (Piao et al. 2007; Xia et al. 2014; Finzi et al. 2020). Measuring the sensitivity of central North American forests to the range of temperatures produced by internal and forced variability is key to predicting how forests will respond to further anthropogenic warming.

In the absence of long ecological records, natural archives can be used to reconstruct past ecosystem processes (West et al. 2006; Evans et al. 2013). Tree rings represent an important source of information on forest growth responses to climate variability over decades to centuries of time (Babst et al. 2013, 2014). Tree-ring analysis, or dendrochronology, can be used to interpret the impacts of a changing climate on forest health and growth (Babst et al. 2014; Klesse et al. 2016; Wilmking et al. 2020). This study predicts that trends in spring temperatures will be reflected in tree-ring width records, as the longer growing season and greater number of days of photosynthetic activity lengthen the period of ring-width formation and lead to wider rings.

In order to scale estimates of forest growth sensitivity from the site-level to a regional basis, satellite observations can tie the results of field studies to remotely detected observations of forest growth (Babst et al. 2018; Seftigen et al. 2018). The most commonly used satellite index used to evaluate ecological changes over time is the Normalized Difference Vegetation Index (NDVI) (Tucker and Sellers 1986; Myneni et al. 1997; Tucker et al. 2001; Kerr and Ostrovsky 2003; Pettorelli et al. 2005; Huang et al. 2021). Because NDVI follows a seasonal trajectory, previous studies have used threshold approaches wherein certain values correspond to the start of biological spring (White et al. 2014; Wang et al. 2018; Kern et al. 2020). These threshold dates are phenological indicators that can be evaluated with respect to their climate sensitivity and compared directly with field observations (Kaufmann et al. 2008; Bunn et al. 2013; Seftigen et al. 2018). In this study, tree-ring data and satellite observations were combined to measure the sensitivity of forest growth and canopy phenology to spring temperatures variability.

### Detecting regime shifts in climate and ecological time series

Whether the warming trend will progress linearly or whether there will be non-stationarity patterns of change that abruptly alter relationships between climate and ecosystems remains highly uncertain (Bueno de Mesquita et al. 2021). A method of identifying changes in climate time series is to model the presence of statistical departures from historical baselines (Reeves et al. 2007; Wilmking et al. 2020). Identifying regime changes can reveal links between major phase changes in atmospheric circulation and regional climatology and ecosystem function. In this study, a class of Bayesian model known as a Hidden Markov Model (HMM) was implemented to detect state changes in spring temperature and tree-ring records (Evin et al. 2011; McClintock et al. 2020). HMMs provide the ability to model transitions between states in time series by classifying the data into distinct regimes that can be evaluated with respect to major shifts in climate drivers (Mallya et al. 2013; Gennaretti et al. 2014).

### Research objectives and hypotheses

The objective of this research was to evaluate variability and trends in spring (March-April-May) temperatures across the Great Lakes region and to analyze the influence of changing spring climate on forest phenology and annual growth. In order to achieve this, (1) local temperature time series were compared with models of external (anthropogenic) forcing and decadal-scale modes of variability. (2) Abrupt shifts in time series data were identified and evaluated with respect to global warming and large-scale modes of atmospheric circulation. Finally, (3) dendrochronology and remote sensing were leveraged to determine the sensitivity of forest growth and phenology to variability and trends in spring temperatures. We hypothesized (H1) that regional climate will exhibit a warming trend consistent with the rate of external forcing from greenhouse gas emissions, but that interannual variability will be strongly correlated with hemispheric patterns of atmospheric circulation, (H2*)* that the rapid rate of warming has led to a state-change in the regional temperature regime, and (H3) that long ecological records will show similar trends and state changes to the regional temperature record.

## Methods

### Analysis of regional climatology

A variety of different datasets were employed to characterize change in regional climate and ecosystem dynamics (Table 1). At the site-level, weather station data from two research sites in northern Minnesota—the Cloquet Forestry Center (CFC) and Marcell Experimental Forest South station (MEF) (Gill 2020; Sebestyen et al. 2020, 2021)—were analyzed. Both stations record temperature and precipitation and provide continuous daily coverage for the period from 1911–present and 1961–present, respectively. Station data at both sites conform to high standards of meteorological data quality and are part of the NOAA Global Historical Climatology Network which conducts both automated quality assurance and works with station managers to ensure high-quality data (Durre et al. 2010; Menne et al. 2012).

**Table 1:** Complete list of datasets used in this study. CFC, Cloquet Forestry Center; MEF, Marcell Experimental Forest; CRU TS, Climate Research Unit Time Series; PDSI, Palmer Drought Severity Index; CMIP6, Coupled Model Intercomparison Project; PNA, Pacific North America Pattern Index; NAM, North Annular Mode Index; MODIS, Moderate Resolution Imaging Spectrometer Normalized Difference Vegetation Index. Additional information on tree-ring chronologies is included in Table S2

Dataset	Time period	Geographic extent	Data type
CFC station	1911–2021	46.70 N, −92.52 W	Daily temp, precip
MEF station	1961–2021	47.57 N, −93.48 W	Daily temp, precip
CRU TS 4.06	1901–2021	−97.8 W, −80.3 W, 40.3 N, 52.8 N	Monthly temp
PDSI	1901–2014	−97.8 W, −80.3 W, 40.3 N, 52.8 N	Monthly soil moisture
CMIP6 ensemble mean	1850–2014	−93.5 W, −92.5 W, 47.0 N, 47.5 N	Monthly temp
CMIP6 piControl	1850–2550	−93.5 W, −92.5 W, 47.0 N, 47.5 N	Monthly temp
PNA index	1950–2021	Northern hemisphere	Monthly indices
NAM index	1900–2021	Northern hemisphere	Monthly indices
Tree ring data (five sites)	Variable	At CFC and MEF site locations	Annual ring width
MODIS NDVI	2003–2021	−96.0 W, −82.0 W, 42.0 N, 50.0 N	Daily NDVI

A suite of gridded data of both temperature and precipitation were analyzed, all available through the University of East Anglia Climate Research Unit (CRU) (Harris et al. 2020). All CRU datasets are on a 5°x5° grid, with a monthly temporal resolution and cover the period from 1901 to 2020. The CRU Time Series (TS) 4.06 monthly gridded climate datasets for temperature and precipitation were used, as well as the CRU Self-calibrating Palmer Drought Severity Index (scPDSI) dataset (van der Schrier et al. 2013; Harris et al. 2020). The region encompassing 80.25W to 96.75W longitude, and 40.25N to 52.75N latitude was included in the analysis. All climate variables were analyzed in this study, but given the emphasis on spring climatology, results for precipitation and other seasonal climate variables are reported in supplemental materials (Fig. S1). Single grid cells for the nearest to the weather stations were extracted for comparison, and gridded data were used to evaluate spatial patterns of change across the Great Lakes region.

The attribution of external anthropogenic forcing on regional climate change was done using an ensemble mean of 33 models contained within the Coupled Model Intercomparison Project Phase 6 (CMIP6) archive representing both anthropogenic and natural forcing for the historical period from 1850 to 2014 (Eyring et al. 2016) (list of models provided in Table S1). Single model grid cells nearest to the weather stations were extracted for comparison, and given that the pixels were very large, both sites were represented by a single pixel. For comparison with the instrumental record, only the modeled period from 1901 to 2014 was analyzed. The ensemble mean was compared with a single, 701 year-long pre-industrial control run representing internal variations only covering the period from 1850 to 2550 (Yukimoto et al. 2019). To produce a distribution of values from the control run of the same length as the overlapping period between station and model data, one-hundred 50-year segments were randomly resampled from the control run, and mean temperature and standard deviations and trend estimates were generated from the resampled data (Karoly and Stott 2006; Dean and Stott 2009). All model data were compared with the gridded and station data by comparing the absolute change, as well as the strength of the decadal-scale trend over the common fifty-year period of 1961–2010 (Dean and Stott 2009; Hegerl and Zwiers 2011).

To ascertain the role of internal climate variability, the PNA and NAM indices were analyzed with respect to their relationships to spring temperatures. Both indices are calculated by taking the leading mode variability from an empirical orthogonal function (EOF) of monthly mean atmospheric pressure (Barnston and Livezey 1987; van den Dool et al. 2000). The NAM index is defined as the first EOF of winter (Dec, Jan, Feb, Mar) sea level pressure across the Northern Hemisphere (20–90°N) from 1900 to 2020 (Thompson and Wallace 1998, 2000, 2001; Thompson et al. 2000, Hurrell and Deser 2009). The PNA index is based on a leading EOF at 500 mb centered over the North Pacific and covers the period from 1950 to 2020 (Trenberth and Hurrell 1994; Yu et al. 2007). For this analysis, mean December–March values of the PNA were analyzed for consistency with the NAM index. The PNA and NAM were spatially correlated with spring (March-April-May) temperatures using the CRUTS 4.06 dataset (Harris et al. 2020).

### Detecting state changes in temperature and ring-width records

**Fig. 1:**
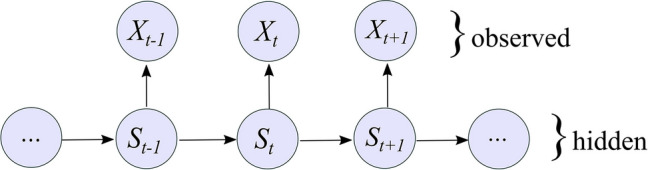
Graphical representation of a basic Hidden Markov Model, in which *X*_t_ refers to the observed temperature or tree-ring records and *S*_t_ refers to the underlying climate state

To test for state changes in climate and associated changes in ecological time series, Hidden Markov Models (HMMs) were used (Evin et al. 2011; Gennaretti et al. 2014). These models have been used in climatology (Evin et al. 2011; Mallya et al. 2013; Gennaretti et al. 2014) and are gaining popularity in ecology and population dynamics (Langrock et al. 2012; McClintock et al. 2020). The advantage of this type of model is that it makes few prior assumptions regarding the timing of different transitions and yields a set of posterior probabilities of the likelihood of regime change, which can be used to ascribe confidence estimates to modeled states. The basic structure of an HMM can be represented as a series of observations determined by underlying hidden states (Fig. 1):

The initial model distribution and parameters can be tailored to fit the data itself. In this case, models were defined following the mathematical formula:

$$p\left({X}_t|{S}_t=k\right)=N\Big({X}_t\mid {\mu}_k,{\sigma}_k^2\Big)$$

**(1)**

where *p* is the probability that observation *X* falls into state *S*_*1*_ or *S*_*2*_ at time step *t*, where *k* describes an initial distribution, in this case a Gaussian function (${\mu}_k,{\sigma}_k^2$). At each time step, the conditional distribution of the observation given a climate state *P(X*_*t*_*|S*_*t*_*)* is dependent on the previous climate state *P(S*_*t*_*|S*_*t−1*_*)* following a Markov process (Mallya et al. 2013)*. S*_*t*_ is defined by a state-dependent distribution (*k)* which is unique to *N* number of different states. The probability of switching between state *S* at time *t* to state *S+1* at time *t+1* is referred to as the transition probability, which is represented using a matrix with *N,* in this case 2, dimensions.

$$\boldsymbol{P}\left({\boldsymbol{x}}_{\boldsymbol{t}}\right)=\left[\begin{array}{cc}\boldsymbol{f}\left({\boldsymbol{x}}_{\boldsymbol{t}}|{\boldsymbol{S}}_{\boldsymbol{t}}=\textbf{1}\right)&\ \textbf{0}\\ {}\textbf{0}&\ \boldsymbol{f}\left({\boldsymbol{x}}_{\boldsymbol{t}}|{\boldsymbol{S}}_{\boldsymbol{t}}=\textbf{2}\right)\end{array}\right]\genfrac{}{}{0pt}{}{{\boldsymbol{S}}_{\boldsymbol{t}}=\textbf{1}}{{\boldsymbol{S}}_{\boldsymbol{t}}=\textbf{2}}$$

**(2)**

The initial transition matrix for the Gaussian model specifies a high probability of being in the first state at the beginning and a low probability of switching between states at each time step, making it conservative with respect to predicting state changes. The Markov process, implemented by a forward algorithm through the depmixS4 R package, calculates the step-by-step dependency of each observation on the previous one to determine the likelihood of transition at each time step (Zucchini and MacDonald 2009; Visser and Speekenbrink 2010). In order to determine whether the transitions between states were conditioned by other variables, co-variates for relevant predictors were added to the transition matrix to model the relationship among states. The Akaike Information Criterion (ΔAIC), Bayesian Information Criterion (BIC), and log-likelihood ratio tests were used to compare model performance between the one- and two-state models (Gotelli and Ellison 2013).

The data that were analyzed for the presence of major state changes in climatology were the mean spring temperature time series from both meteorological stations and the nearest pixel from the CRU data for both meteorological site locations. Three models were fit to each time series: a null model with only one state, a simple two-state model with no covariates, and a full model with multiple covariates on the transition probabilities. The CMIP6 ensemble mean curve and atmospheric circulation datasets were included in the full model as covariates. Standardized tree-ring width data from both site locations were also modeled using HMM models for the presence of state changes. Three models were fit in the case of the tree-ring data; a null one-state model, a simple two-state model with no covariates, and a full model with spring temperature (from the CRU dataset) fit as a covariate. In order to identify transition years, the posterior states were examined, and if a single transition year was present in the data, this was reported as a transition year.

### Tree-ring data development

**Fig. 2:**
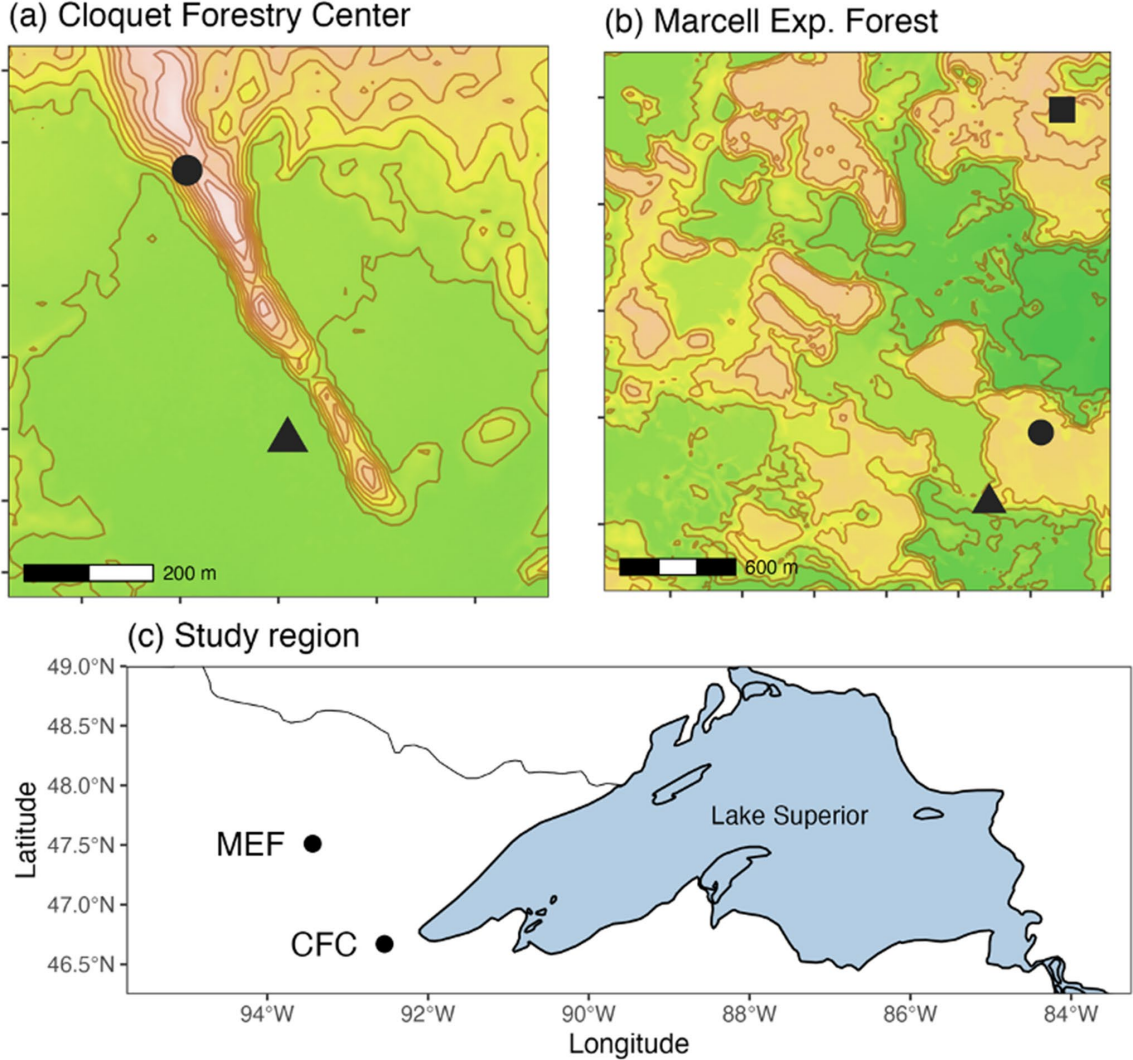
Research sites where sampling took place, with locations and species of original tree-ring datasets indicated. **a** Cloquet Forestry Center (CFC) with two tree-ring sites indicated. **b** Marcell Experimental Forest (MEF), the location of three study sites. Triangles indicate black spruce (*Picea mariana*) sites, circles represent red pine (*Pinus resinosa*), and squares represent eastern Larch (*Larix laricina*) sites. Contour lines are draw at 3-m intervals from a LiDAR-based digital elevation model sourced from the MN DNR MNTopo program, and geographic and hydrologic boundaries are from the Natural Earth database. **c** Geographic locations of research sites relative to the greater region

Tree-ring data co-located with local weather stations were collected at two sites in northern Minnesota (Fig. 2). The Cloquet Forestry Center (CFC) (46.70 N, −92.52 W) (Fig. 2, a) was established in the 1909 by the University of Minnesota Department of Forest Resources as an experimental research forest. The Marcell Experimental Forest (MEF) (47.57N, −93.48) (Fig. 2, b) was established by the USDA Forest Service in the 1960s to study the hydrology and biogeochemistry of forested peatlands (Kolka et al. 2011). Both sites represent managed, mixed hardwood and conifer forests on heterogeneous topography with soils ranging from well-drained sandy uplands to peat-dominated lowlands (Kolka et al. 2011; Gill et al. 2022). At CFC, black spruce (*Picea mariana*) and upland red pine (*Pinus resinosa*) were sampled. At MEF, red pine, black spruce, and Eastern larch (*Larix laricina*) were cored. Each site represents fifteen individual trees, with two increment cores collected per tree.

### Tree-ring chronology development and climate response analysis

Standard dendrochronological methods were used to develop site chronologies for all five sampling locations across both research forests. Cores were dried, mounted on wood blocks, and sanded using successively finer-grit sandpaper, through to a 1000-grit polishing paper. Cross-dating and total-ring width measurements were done using a Velmex measurement system (Velmex Inc. 2009). Dating accuracy was assessed using the COFECHA cross dating software to identify and resolve dating issues (Holmes 1983). Expressed population signal (EPS) and RBAR were used alongside statistical cross dating to determine the strength of coherence among series in the chronologies, with an EPS cutoff of 0.85 used to determine whether the chronology expressed strong agreement across series (Table S2) (Wigley et al. 1984). Average chronologies of dimensionless ring width indices were calculated using the dplR package in the R statistical programming environment (Bunn 2008). Ring-width indices were detrended using site-specific detrending choices, either using negative exponential curves or 100-year splines, depending on the site. Due to stand-wide disturbances, many ring-width series exhibited periodic growth releases in the middle of the series, such that negative exponential curves were not appropriate in all cases and rigid splines were preferred in most cases (Fig. S2).

Simple correlation analysis on the mean chronologies determined the relationship of annual growth to a variety of climate variables (Fig S1). To determine the changing relationship of tree-growth and spring temperature, running Pearson’s correlation analyses was used on the climate-growth relationships, binned by decade. Running correlations were done using the CRU TS 4.06 data. Running correlations were calculated over 10-year periods using the gtools package (Bolker et al. 2022), and statistical significance of moving correlations was determined using a Mann-Kendall test for trends with a block bootstrapping to determine a null distribution of correlation values and to assess significance and derive confidence limits (Önöz and Bayazit 2012; Kokfelt and Muscheler 2013). One thousand iterations were run on each time series, segmented by 10-year blocks.

### Remote detection and analysis of canopy phenological responses to spring temperature

To track the sensitivity of canopy phenology to spring temperatures, the Moderate Resolution Imaging Spectrometer (MODIS) daily Normalized Difference Vegetation Indices (NDVI) from 2003 to 2021were analyzed. These indices are produced using 16-day return images from the MODIS Terra/Aqua Daily Level 3 Global 500-m top-of-atmosphere irradiance images, from which daily surface reflectance composites have been produced that have been masked for cloud cover and corrected for atmospheric conditions (Vermote 2021). The data are provided by the United States Geological Survey and the NASA Earth Data program and accessed using the Google Earth Engine platform (Gorelick et al. 2017). Daily NDVI data are calculated for each pixel using the ratio of a near-infrared and infrared band, at wavelengths that capture major changes in the reflectance spectrum of green vegetation (Kriegler et al. 1969).

**Fig. 3:**
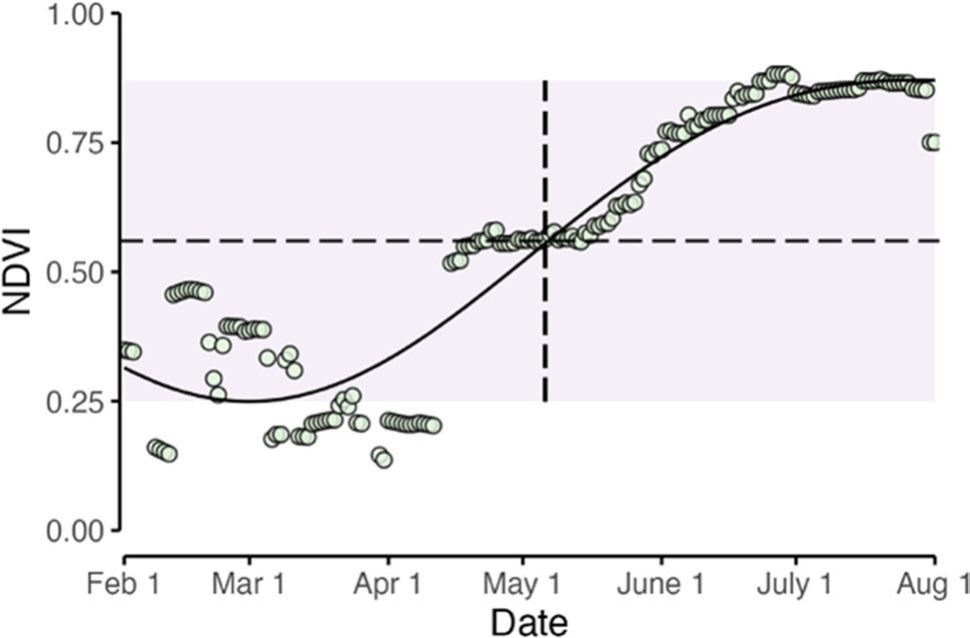
Example NDVI time series, fitted with a harmonic curve for one pixel from northern Minnesota, from the Marcell Experimental Forest, in the year 2009. The NDVI time series is indicated in green circles. The horizontal dashed line indicates the 50% value between the minimum- and maximum-modeled values, and the vertical dashed line indicates the date at which the 50% value was reached. The grey-shaded area reflects the span of NDVI values between modeled min and max

In order to create a model that could capture differences among cover types based on their unique phenological properties, each years’ time series for each pixel was fit with a unique harmonic curve function (Fig. 3). Harmonic curves, based on Fourier-transformed time series, represents each series as a wave defined by the unique amplitude and phase angle (Jakubaukas and Legates 2000). Thus, each curve has a peak, trough, and rate of change that reflect the unique phenological properties of that forest type (Kern et al. 2020; Wang et al. 2018). The method also forces potentially noisy data to conform to a theoretical model in which NDVI is lowest at the beginning of the growing season and peaks in late spring or summer. More information about harmonic curve fitting can be found in Shumway and Stoffer (2017).

Based on the harmonic curve fit for Julian days 1–212 (Jan 1 through the of end of July), a threshold of 50% (hereafter referred to as NDVI_50_) was used as a threshold to establish the start of spring for each MODIS pixel, calculated as the midpoint on the curve between the min and max for each year:

$${NDVI}_{50}={NDVI}_{min}+\left(\left({NDVI}_{max}-{NDVI}_{min}\right)\ast 0.5\right)$$

(3)

Using CRU TS 4.06 gridded temperature record for the spring (MAM) months, point-by-point Pearson’s correlation coefficient was calculated at the 500-m square MODIS pixel size for the correlation between NDVI_50_ and temperatures for the 2003–2021 period. This resulted in a single image of correlation values that could be segmented according to forest cover type. Using a high-resolution forest cover map from the USDA Forest Inventory and Analysis program resampled to the same resolution (500 m) as the MODIS data, each pixel of correlation values were classified by forest type (USDA Forest Service 2008), and then further aggregated into four rough forest type categories represented in the region: upland conifer, lowland conifer, oak savannah, and upland deciduous (Table S2). The sensitivity of different forest types was assessed using linear regression with temperature, with sensitivity to warming indicated by the regression coefficient (α).

## Results

### Contribution of internal and forced variability to trends in regional climate

**Fig. 4:**
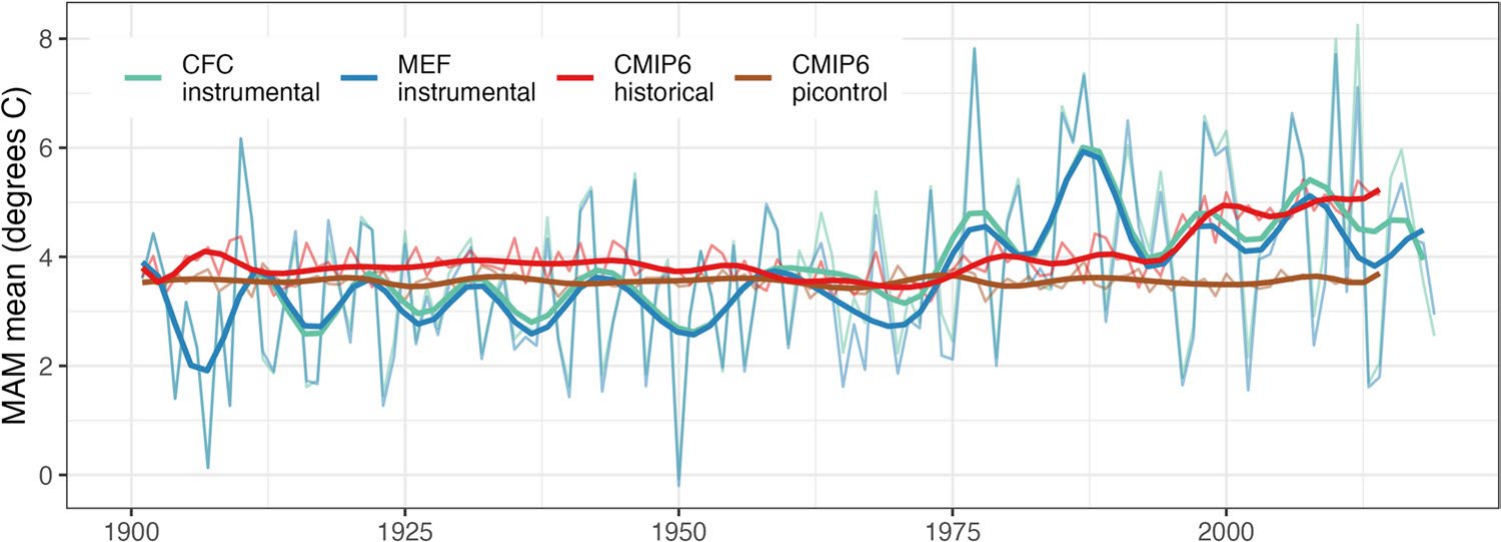
Change in spring temperatures for the Cloquet Forestry Center (CFC) and Marcell Experimental Forest (MEF) compared with (1) a CMIP6 ensemble of historical model runs with all forcing included and (2) a CMIP6 pre-industrial (piControl) run representing only internal variations in climate. piControl-run model data represent an average of resampled data from the 701 year-long full model run. Site-level instrumental data were derived from averaging station and CRU records together for a cleaner visualization. CRU data begin in 1901 and station data begin in 1911 for CFC and 1961 for MEF, years prior are represented by CRU data only. Time series have been fit with a local polynomial spline with 25 degrees of freedom

Results from the attribution study indicated strong agreement between the CMIP6 historical model ensemble mean and local temperature records (Fig. 4, Table 2). Estimates of total warming over the 50-year overlapping period among all station, gridded, and modeled datasets (1961–2010) ranged between 1.5 and 2.1 °C depending on the record, and both stations yielded higher estimates of warming than the CRU-gridded data. The CMIP6 ensemble mean fell in the middle of the range of estimates for rate of warming compared with gridded and station data. However, the CMIP6 temperature data had a somewhat higher baseline of around 4.0 °C on average compared with around 3.8 °C for the instrumental data, potentially dampening the rate of warming by comparison. The piControl model run showed slightly lower baseline temperature and exhibited a slight cooling trend overall during this period.

**Table 2:** Results from the comparison between station, instrumental and model data. Pre-industrial control (piControl) model data represent an average from resampling the data over 100 random 50-year sections to represent the same length of the CRU time series. The trend and total warming are calculated for the 50-year period represented by all datasets (1961–2010)

*Temperature dataset*	*Time period*	*Mean (°C)*	*Standard deviation*	*Trend 1961–2010 (°C/decade)*	*Total warming 1961–2010*
*Instrumental*					
CFC station	1911–2020	3.84	1.54	0.334	+1.64
CFC CRU	1901–2020	3.79	1.62	0.311	+1.52
MEF station	1961–2020	3.82	1.76	0.414	+2.03
MEF CRU	1901–2020	3.82	1.62	0.311	+1.52
*CMIP6 model*					
Ensemble mean	1850–2014	4.01	0.51	0.339	+1.66
PiControl	1850–2550	3.56	1.44	−0.01	−0.05

**Fig. 5:**
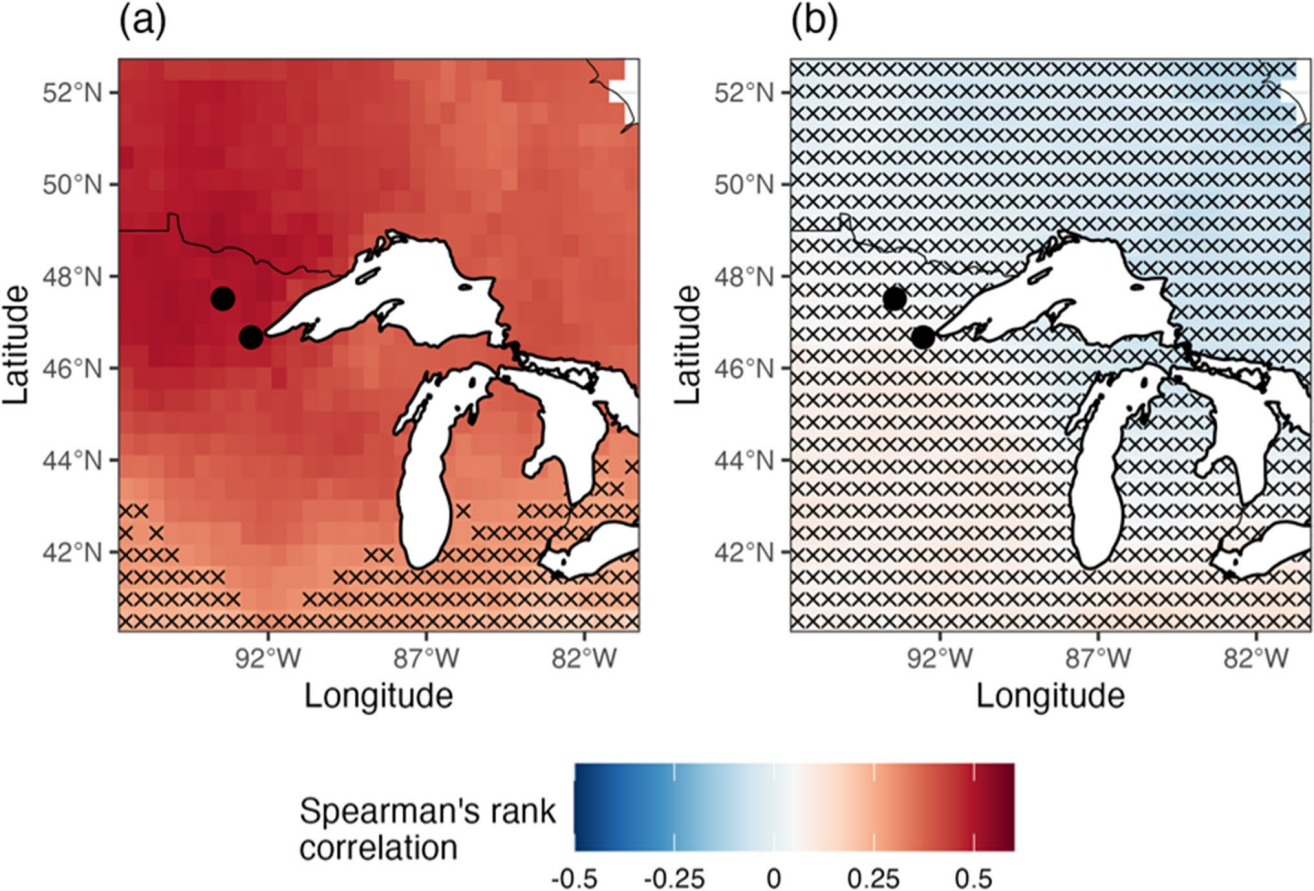
Relationship of winter multidecadal variability to spring temperatures. The **a** Pacific-North American pattern (PNA) and **b** (NAM) Northern annular mode correlation spring temperature across the Great Lakes and upper Midwest regions. Spearman’s rank correlation test was used, with 95% confidence level indicated with black hatching. Grid cells with black x’s are not significant. The two tree-ring study sites are shown as black dots

Analysis of two different modes of winter ocean-atmosphere climate variability indicated that teleconnections to the North Pacific strongly affect spring temperatures across this region (Fig. 5). The PNA, which has been shown elsewhere to influence spring temperatures across this region (Yu et al. 2014), is here shown to be an important driver of spring climatology in the continental interior (Fig. 5a). The influence of the PNA pattern appears here to extend to the eastern edge of the Great Lakes region, with the two tree-ring study sites located with the area most strongly influenced by PNA variability. By contrast, the NAM did not show strong correlations with spring temperature variability (Fig. 5b). Because of the strong relationship between the PNA and temperature, the PNA index is used to model state changes in regional temperature (below) where the NAM is not investigated further.

### Hidden Markov modeling of spring temperatures

The HMM procedure performed over the full period of record identified regime shifts in local climatology at both site locations for all records with the exception of the short MEF station record (Table 4). The state change is indicated by a decrease in AIC, BIC, and a significant difference in log-likelihood. Evaluation of the model posteriors indicated that the shift from the first to second state was stable and that a transition occurred after 1976. The addition of two covariates (the warming curve from the CMIP6 ensemble mean and the time series representing the PNA) to the models reduced the length of the time series in most cases from over 100 years to just over 50 years. The shorter time series presented with greater stochasticity and only in one case was the same 1976 state change identified as a single, stable transition year. In other cases, the models had the tendency to shift between states (Table 3). However, the addition of covariates improved model performance somewhat by decreasing model AIC and resulting in a marginally significant difference in log-likelihood (*p* < 0.1).

**Table 3:** Hidden Markov Model results for regime change in spring climatology at the Cloquet Forestry Center (CFC) and Marcell Experimental Forest (MEF) for both station and Climate Research Unit (CRU) climate data. Model performance for the one-state and two-state model was evaluated by the change in Akaike Information Criterion (Δ AIC). Model log-likelihood is also indicated for all models and is compared among models using a log-likelihood test (**•** = *p* < 0.1, *p < 0.05, **p < 0.01). The transition year (T) is indicated if a single transition year was identified. The superior model is in bold

Record	n state = 1			n state = 2			Model selection	
	AIC	BIC	Log L	AIC	BIC	Log L	Δ AIC	T Year
CFC station	385.7	390.97	−190.85	**374.07**	**392.52**	−180.04**	−11.63	1976
MEF station	221.62	225.6	−108.81	224.63	238.56	−105.32	3.01	–
CFC CRU	438.54	444.01	−217.27	**423.06**	**442.21**	−204.53**	−15.48	1976
MEF CRU	452.89	458.36	−224.44	**444.66**	**463.81**	−215.33**	−8.23	1976
Models with CMIP6 + PNA covariates (1961–2014)								
Record	n state = 2			n state = 2 + covariates				
	AIC	BIC	Log L	AIC	BIC	Log L	Δ AIC	T Year
CFC station	259.45	274.67	−122.72	**258.17**	**277.74**	**−120.08 •**	−1.28	1976
MEF station	**224.63**	**238.56**	**−105.32**	226.42	244.32	−104.21	1.79	–
CFC CRU	261.97	277.19	−123.99	**260.45**	**280.02**	**−121.22 •**	−1.52	–
MEF CRU	273.95	289.17	−129.98	**273.32**	**292.89**	**−127.66 •**	−0.63	–

### Characteristics of the 1976 shift in spring temperatures

**Fig. 6:**
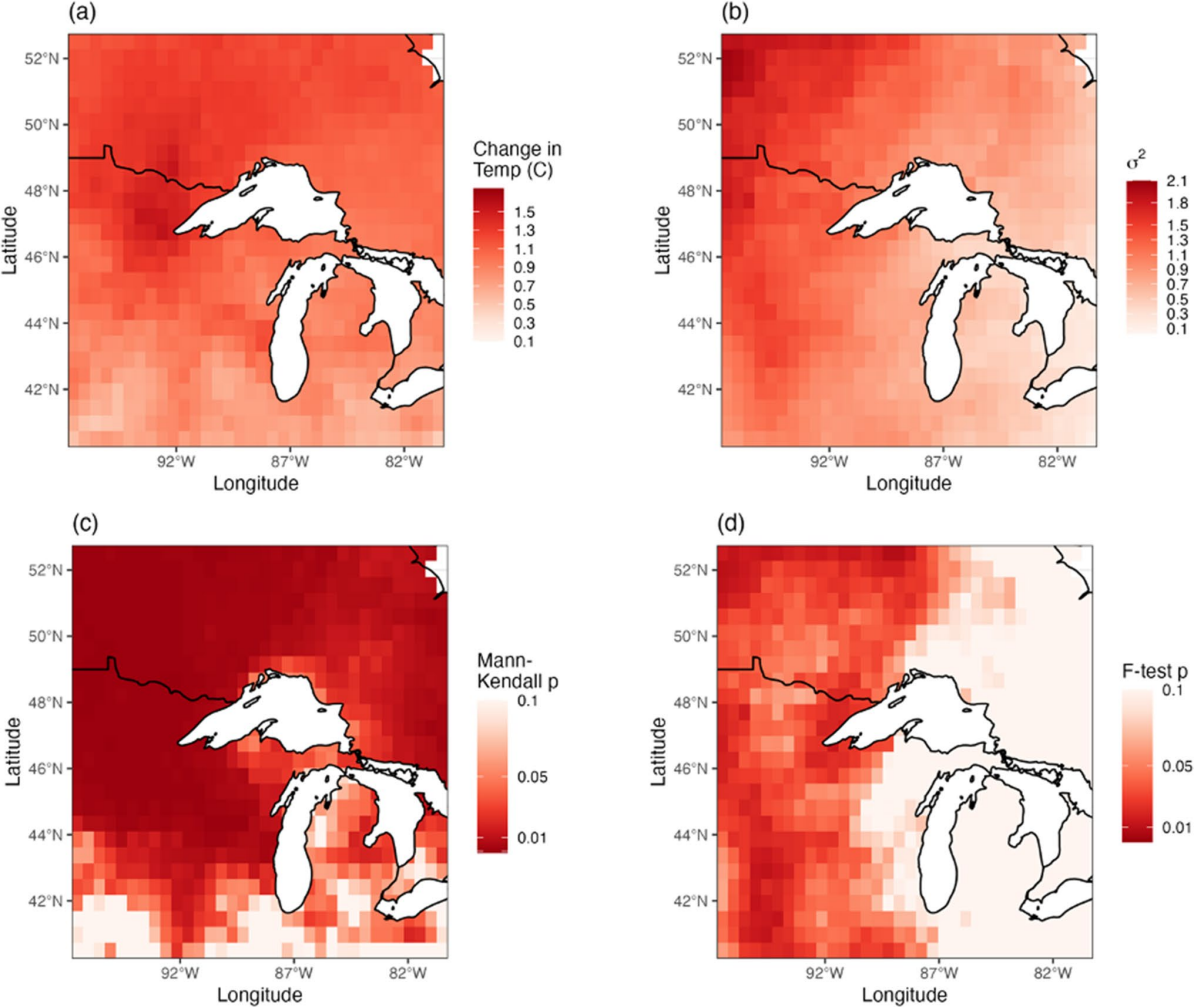
Changes in the mean and variance in spring temperatures and their significance. Clockwise from top left: **a** difference in the mean (pre- and post-1976), **b** change in the variance (pre- and post-1976), **c** Mann–Kendall trend test for entire time series, and d *F* test for significant difference in variance (pre- and post-1976)

Analysis of change in means and variance demonstrate that the local climate at both the CFC and MEF indicates a change in the mean state of climate following 1976. Results of the Mann-Kendall indicate that the warming trend is highly significant in all time series (*p < 0.001)* (Table 4), except the MEF station data which was likely due to the shorter period of record (1961–present). *F* tests comparing pre- and post-1976 variance in the data were marginally or non-significant, indicating that the HMM model result is likely being driven more by changes in the mean values than the variance across years. In particular, the records indicate that spring 1977 was substantially warmer than any previous year (Fig. 3), which is likely why 1976 was consistently identified by the models as a state change year. On a regional basis, examination of changes in mean and variance of temperatures indicated that the region has experienced an increase in average temperatures (Fig. 6, left panels), with a significant increase in variance (Fig. 6, right panels). Increase in average temperatures across the region was approximately 1.5 °C, and significance tests on the regional data indicate that the trend in mean temperatures was significant at a 95% confidence level or greater for the entire region.

**Table 4:** Change in mean and variance at the Cloquet Forestry Center (CFC) and Marcell Experimental Forest (MEF) in the pre- and post-1976 time periods, as well as the results of the Mann–Kendall test for the significance of trends in time series data, and *F* test to test differences in variance between the two time periods. Mann–Kendall and *F* test *p*-values and test statistics indicate model significant and fit

	Pre-1976		Post-1976		Mann–Kendall		*F* test	
*Record*	*Mean °C*	*Variance*	*Mean °C*	*Variance*	*P*	*T*	*P*	*F*
CFC Station	3.33	1.33	4.60	3.00	<0.001	0.230	0.14	0.79
CFC CRU	3.21	1.60	4.78	2.83	<0.001	0.282	0.10	0.78
MEF Station	2.70	1.54	4.23	3.01	0.469	0.001	0.08	0.72
MEF CRU	3.21	1.06	4.78	2.89	<0.001	0.282	0.80	0.96

### Sensitivity of regional forests to spring temperature

**Fig. 7:**
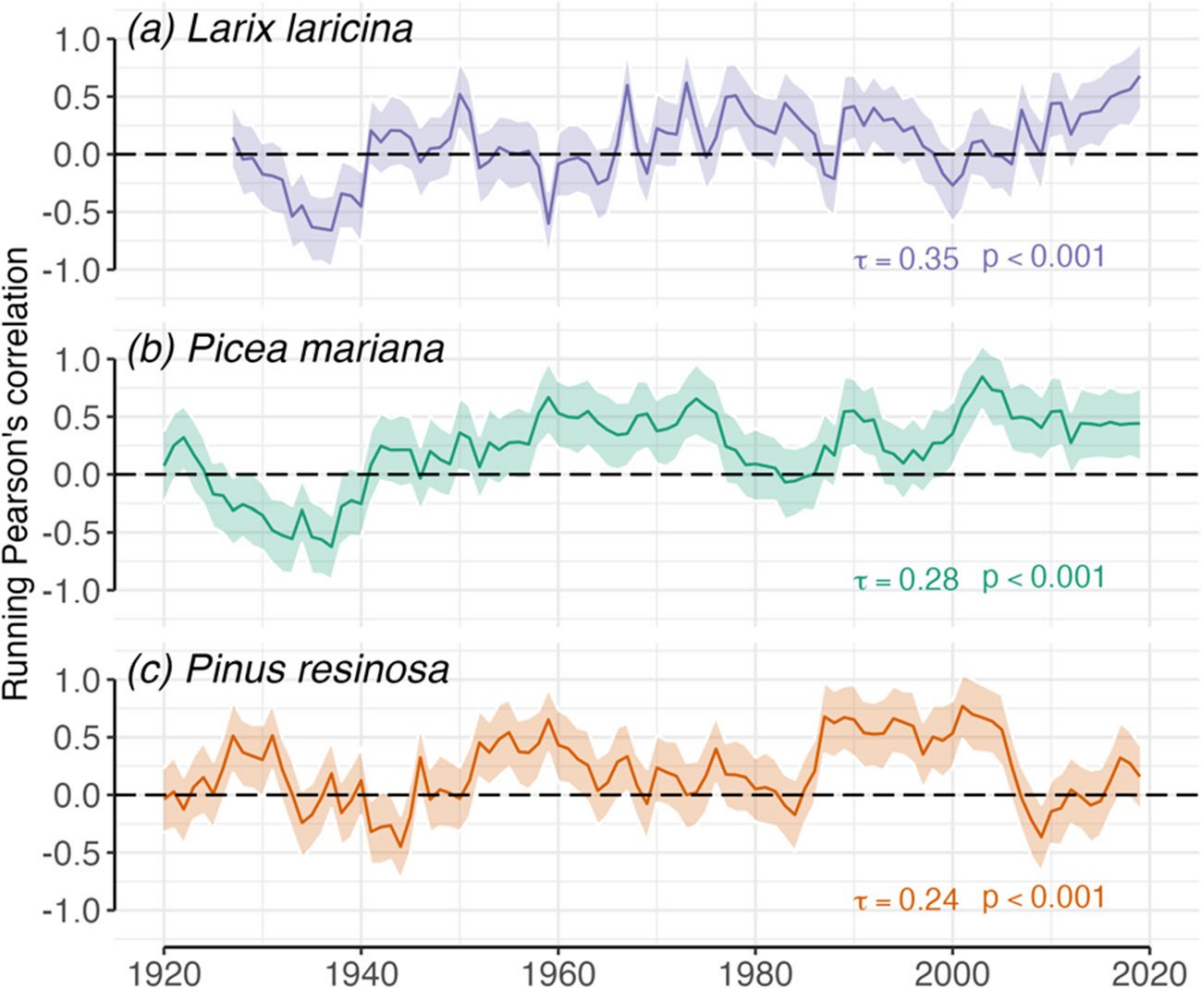
Results of the running correlation analysis of the relationship between spring temperature and tree-ring indices. **a** *Larix laricina* (one site), **b** *Picea mariana* (2 sites), and **c** *Pinus resinosa* (2) sites. Colored lines represent running correlations between decadally averaged ring width and temperature, with a 90% confidence threshold indicated by the black dashed horizontal line. The significance of the change in sensitivity was established for each species using a non-parametric Mann-Kendall test for the significance of autocorrelated time series, with block-bootstrapping with 10-year blocks. Model *p*-values and test statistics (T), as well as the slope and shaded confidence intervals, are derived from the bootstrapped models

Results from the analysis of the relationships between climate and tree growth in northern Minnesota showed a significant positive response to spring temperatures (Fig. 7). The 10-year running correlation analysis indicated that the relationship between growth and spring temperatures increased significantly over time, but that the majority of individual years were not significant when evaluated against a null distribution simulated using a Monto Carlo method. For both lowland species, the correlation started around zero and strengthened the most over the course of the period of record.

The HMM models, applied to the tree-ring data, also indicated sharp breakpoints in the tree-ring records—evidence that non-stationarity of the tree-ring chronologies (Table 5). In all cases, the two-state model was superior to the null (one-state) model, and in two cases, adding a covariate for temperature improved model performance as evaluated through change in AIC and log-likelihood ratio testing. Visual examination of the posterior probability time series indicated that the two-state models were unstable through time and highly likely to shift back and forth between states.

**Table 5:** Hidden Markov Model results for tree ring indices, with spring temperature from the CRU record added to the model as a covariate. Model results are included for the Cloquet Forestry Center (CFC) and Marcell Experimental Forest (MEF) for three species, *Pinus resinosa* (PIRE), *Picea mariana *(PIMA), and *Larix laricina *(LALA). Model performance for the one-state, two-state, and two-state + covariate models were evaluated by the change in Akaike Information Criterion (Δ AIC and Δ BIC). Model log-likelihood is also indicated for all models and is compared among models using a log-likelihood ratio test (**•** = *p* < 0.1, *p < 0.05, **p ≤ 0.01). The superior model is indicated in bold

Models with no covariates								Models with CRU temperature covariate			
	n state = 1			n state = 2			Model selection	n state = 2 + temp			Model selection
Record	AIC	BIC	Log L	AIC	BIC	Log L	Δ AIC	AIC	BIC	Log L	Δ AIC
CFC PIMA	**−**90.73	**−**85.25	47.36	**−122.58**	**−103.43**	68.29**	**−31.85**	**−**121.69	**−**97.07	69.85	0.89
MEF PIMA	**−**32.27	**−**26.8	18.14	**−**56.49	**−**37.33	35.24	**−**24.22	**−59.3**	**−34.67**	38.65*	**−2.81**
CFC PIRE	**−**88.66	**−**83.19	46.33	**−115.22**	**−96.07**	64.61**	**−26.56**	**−**113.75	**−**89.12	65.87	1.47
MEF PIRE	**−**15.65	**−**11.24	9.83	**−20.17**	**−4.74**	17.09**	**−4.52**	**−**16.25	3.59	17.13	3.92
MEF LALA	113.29	118.44	**−**54.65	82.91	100.93	**−**34.45	**−**30.38	**77.57**	**100.74**	-29.79**	**−23.36**

### Sensitivity of canopy phenology to spring temperatures

**Fig. 8:**
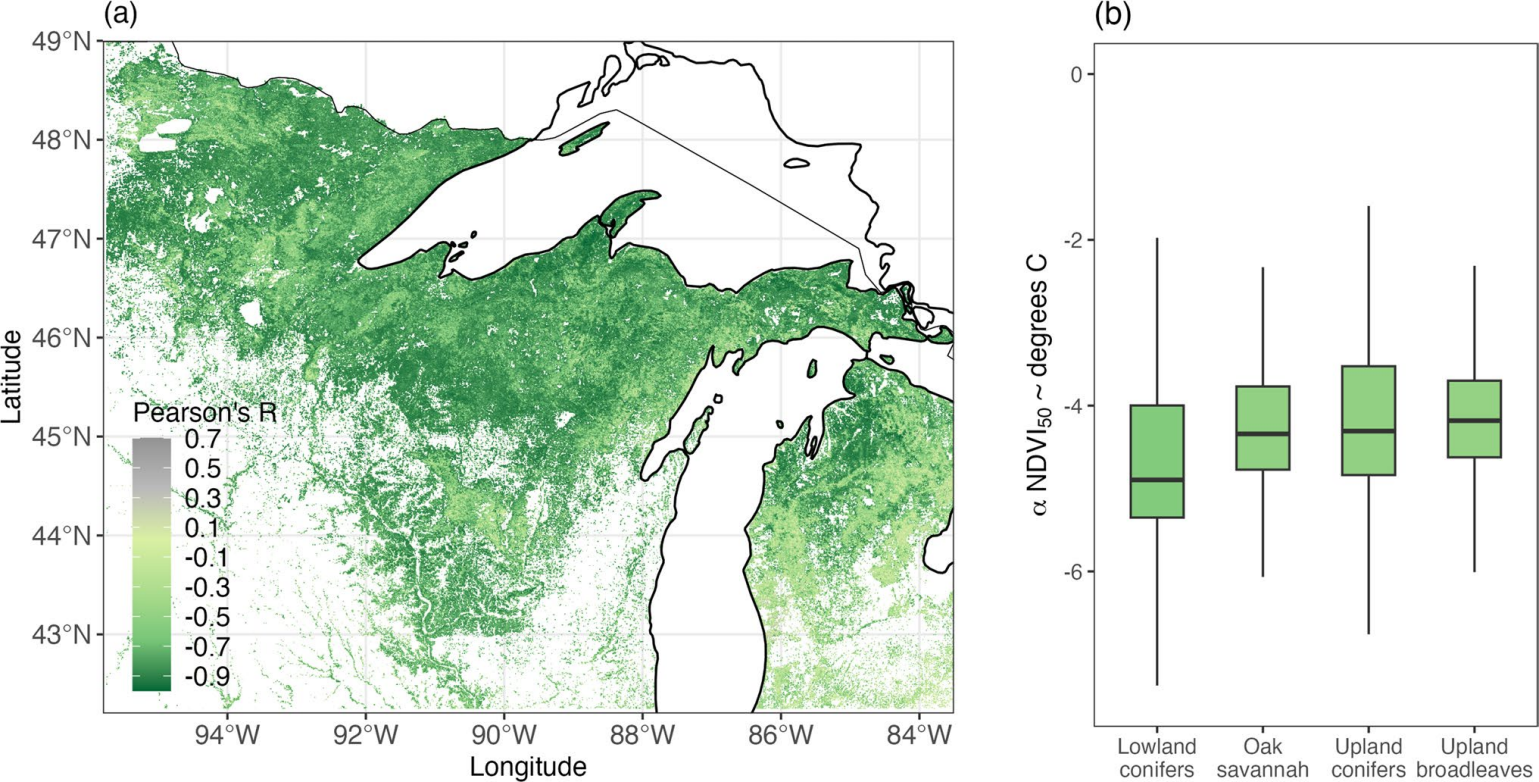
Correlation between date at which NDVI for forested pixels achieved a threshold value of 50% of peak and average spring temperatures (in degrees Celsius) from 2002 to 2021. Analysis was done on a point-by-point basis between 500 m^**2**^ MODIS pixels and 0.5-degree CRUTS 4.06 temperature data. **a** Map showing spatial patterns of negative correlation between average (monthly) spring temperatures and forest phenology (measured in change in NDVI by Julian day). **b** Histogram showing the variation in sensitivity to spring temperature variability by cover type. Boxplots indicate the regression coefficients from a linear model describing the sensitivity of green-up to change in temperature

The correlation between forest canopy phenology and spring temperatures was strong across the region, varying from −0.3 to −0.9, with an average correlation value of 0.69 for the entire domain (Fig. 8a). Classifying the pixels by cover type yielded differences by category, with lowland conifers in general showing more sensitivity than broadleaves (Fig. 8b).

## Discussion

The timing of the transition from winter into spring has far-reaching implications for society and nature. In central North America and the Great Lakes, spring temperatures affect agricultural production and ice-out dates for lakes shipping routes (Millerd 2011; Hatfield 2020). The timing of warm weather also has cascading effects on the function and productivity of terrestrial ecosystems (Cleland et al. 2007; Polgar and Primack 2011; Vitasse et al. 2009). Phenological mismatches between species that rely on each other for food or pollination have negative consequences for the reproduction and survival of both plants and animals, restructuring ecosystems over time (Inouye 2008; Polgar and Primack 2011; Kudo and Ida 2013; Renner and Zohner 2018). The lengthening of the growing season can also affect net primary production (NPP), although the effects of the advance in spring on whole ecosystem carbon budgets appears to vary substantially by forest type, species composition, and age structure (Piao et al. 2007; Richardson et al. 2009; Hänninen 2016; Dow et al. 2022). In this study, pronounced changes in Great Lakes regional spring temperatures were produced following a major shift in ocean-atmosphere variability, which has been sustained over the past several decades.

Attribution of the warming trend to external forcing indicated strong agreement between the CMIP6 historical ensemble mean and local station and gridded instrumental data (Eyring et al. 2016). Analysis of the CMIP6 ensemble for this region yielded an estimate of regional warming for the overlapping period from 1961 to 2010 of +1.66 °C, which was in range with the instrumental data, (+1.52–2.03 °C). The station data exhibited a higher rate of warming than the CRU data, possibly due to local effects biasing the station records. However, the CMIP data had a higher baseline temperature estimate prior to the start of the period of anthropogenic forcing, such that total magnitude of change in the recent period was reduced. Greenhouse gas emissions account for the total warming fraction in CMIP6 models, so even though the specific forcings (e.g., solar and volcanic forcing, aerosols, greenhouse gases) were not individually examined in the course this study, anthropogenic greenhouse gas emissions are the only factor that could have caused this trend (Tokarska et al. 2020).

Internal climate variability interacts with anthropogenic forcing on interannual to decadal time scales, contributing to uncertainty in regional climate change projections (Maher et al. 2020; Yu et al. 2020). The detection of abrupt departures from historical mean states can reveal information about underlying climatology by relying solely on observational data (Hansen et al. 2016). In this study, 1976 marked a transition from a cooler spring climate into a warmer one. The addition of covariates for external forcing and PNA circulation improved model performance, indicating a contribution of both factors to the observed state change. The winter of 1976/1977 is known in the climate literature as the timing of a regime shift in North Pacific sea surface temperatures (Miller et al. 1994; Mantua and Hare 2002; Bond et al. 2003; Deser et al. 2004; Yeh et al. 2011). The following years were marked by a prolonged warm period across the tropical Pacific basin, which contributed to the maintenance of the PNA positive phase until 1988 (Trenberth and Hurrell 1994). The shift back to a negative phase after 1988 is not reflected in regional climatology, suggesting that the secular warming trend played a role in the recent changes in spring timing (Ault et al. 2015). This highlights the integration of internal and forced variability on decadal time scales and illustrates the crucial role of internal variability in modulating regional temperature regimes.

Tree-ring width chronologies exhibited an increasing sensitivity to spring temperatures over the same period, although visual and statistical examination of the tree-ring time series and model posteriors indicated heterogeneous climate responses and non-linear growth patterns, evidence that warming signals are being modified by other factors such as precipitation or stand-wide disturbances (Thom et al. 2018). In temperate, mesic forests such as those in northern Minnesota, variability in ring widths is often driven by stand dynamics more strongly than by climate (Foster 1988; Rollinson et al. 2021). At multiple sites, evidence of disturbance-related growth releases was found during the twentieth century (Fig. S2). In the lowland sites as a likely effect of draining forested wetlands to increase tree growth rates (Kolka et al. 2011; Gill et al. 2022). A possible extension of this research would be to use an HMM framework to model disturbance-related growth patterns alongside changes in the climate system (McClintock et al. 2020).

Satellite phenological observations indicated strong regional relationships between green-up dates and spring temperatures, consistent with other studies (Richardson et al. 2006; Piao et al. 2007; Schwartz et al. 2012; White et al. 2014). Segmenting by forest type suggested that phenology in lowlands and conifer stands was particularly sensitive to variability in spring temperatures, contrasting with research indicating that broadleaf forests were more sensitive to warming than conifers (Richardson et al. 2006; Montgomery et al. 2020). All of the tree-ring datasets were collected from conifers, and the lowland sites also showed a greater response than the upland sites, across all datasets and analytical approaches. Other studies have made direct comparisons between NDVI and tree-ring width measurements as both are theoretically measurements of forest growth (Kaufmann et al. 2008; Bunn et al. 2013; Seftigen et al. 2018). Experimenting with comparison of site-level NDVI and tree-ring widths did not yield strong relationships for these sites, potentially due to the mixing of cover types within the large satellite pixels (Fisher et al. 2006; White et al. 2014). Despite the challenges of linking tree-ring data directly with satellite records, the results of our analysis suggest a region-wide trend towards earlier leaf-out correlated with higher radial growth rates in conifer-dominated systems.

## Conclusion

Trends in spring temperatures stemming both from anthropogenic forcing and internal climate dynamics exert a combined influence on regional climate and ecosystems over time. This study identified a regime change in the mean state of spring climatology across the Great Lakes region following a major shift in North Pacific atmospheric circulation, which was sustained across subsequent decades due to global warming. The effects of warming were evident in tree-rings as an increase in ring width, and in satellite observations phenology as an advance in the timing of spring greening. In the second-growth forests of the upper Midwest decoupling the effects of internal and forced climate variability from forest management and disturbance regimes require novel, dynamic methods of change detection.

### Supplementary information

ESM 1 (PDF 581 kb)

## Data Availability

All datasets used in this study are publicly-available. The CRU TS 4.05 and 4.06 climate datasets are available on the University of East Anglia Climate Research Unit website (https://crudata.uea.ac.uk/cru/data/hrg/). The Pacific-North America pattern index data used in this study are available are available through the National Oceanic and Atmospheric Administration (NOAA) Climate Prediction Center data clearinghouse, and the Northern Annular Mode data are available through NOAA National Center for Atmospheric Research climate data portal. All satellite data were accessed through the Google Earth Engine Data Catalog, and made available by the United States Geological Survey Land Processes Distributed Active Archive Center. Station data are available on the websites of each site location (references herein). All five tree-ring datasets are publicly-available on the NOAA International Tree-Ring Databank (McPartland 2023). Code will be made available upon request.
