# Supplementary material for: Decadal-scale variability and warming affect spring timing and forest growth across the western Great Lakes region
Source: Int J Biometeorol. 2024 Jan 18;68(4):701–17. doi: 10.1007/s00484-023-02616-y (PMC10963445; doi:10.1007/s00484-023-02616-y)
Supplement: Supplementary file 1 — (PDF 581 kb) [file 484_2023_2616_MOESM1_ESM.pdf]

Supplemental Materials

Title: Decadal-scale variability and warming affect spring timing and forest growth across the western Great Lakes region

Author: Mara Y. McPartland[1,2]

Affiliations: [1]Alfred-Wegener-Institut Helmholtz-Zentrum für Polar- und Meeresforschung, Potsdam, Germany [2] Department of Geography Environment & Society, University of Minnesota, Minnepolis, Minnesota, USA

Email: mara.mcpartland@awi.de

Journal: *International Journal of Biometeorology*

Table S1: List of Coupled Model Intercomparison Project Phase 6 models used in this study. Full references are provided below.

Model	Realization	Institute	Country	Resolution	Reference
ACCESS-CM2	r1i1p1f1	Commonwealth Scientific and Industrial Research Organisation-Australian Research Council Centre of Excellence for Climate System Science	Australia	180 x 80	Mackallah et al. 2021
ACCESS-ESM1-5	r1i1p1f1			181 x 80	Mackallah et al. 2021
AWI-CM-1-1-MR	r1i1p1f1	Alfred Wegener Institute for Polar and Marine Science	Germany	192 x 96	Semmler et al. 2020
AWI-ESM-1-1-LR	r1i1p1f1			192 x 96	Danek et al. 2020
BCC-CSM2-MR	r1i1p1f1	Beijing Climate Center	China	320 x 160	Wu et al. 2019
BCC-ESM1	r1i1p1f1			320 x 160	Wu et al. 2020
CAMS-CSM1-0	r1i1p1f1	Chinese Academy of Meteorological Sciences	China	320 x 160	Rong et al. 2019
CAMS-CSM1-0	r1i1p1f2			320 x 160	Rong et al. 2019
CanESM5-CanOE	r1i1p2f1	Canadian Centre for Climate Modelling and Analysis	Canada	320 x 160	Christian et al. 2022
CanESM5	r1i1p1f1			320 x 160	Swart et al. 2019
CanESM5	r1i1p2f1			320 x 160	Swart et al. 2019
CESM2-FV2	r1i1p1f1	National Center for Atmospheric Research	USA	288 X 192	Danabasoglu eet al. 2020
CESM2	r1i1p1f1			288 x 180	Danabasoglu eet al. 2020

CESM2-WACCM-FV2	r1i1p1f1			288 x 192	Danabasoglu eet al. 2020
CESM2-WACCM	r1i1p1f1			144 x 96	Danabasoglu eet al. 2020
CMCC-CM2-HR4	r1i1p1f1	Fondazione Centro Euro-Mediterraneo sui Cambiamenti Climatici	Italy	288 x 192	Cherchi et al. 2018
CMCC-CM2-SR5	r1i1p1f1			288 x 192	Cherchi et al. 2018
CMCC-ESM2	r1i1p1f1			288 x 192	Cherchi et al. 2018
FGOALS-g3	r1i1p1f1	Chinese Academy of Sciences	China	180 x 80	Li et al. 2020
FIO-ESM-2-0	r1i1p1f1	First Institution of Oceanography	China	288 X 192	Song et al. 2019
GISS-E2-1-G	r1i1p5f1	NASA Goddard Institute for Space Studies	USA	144 x 90	Kelly et al. 2020
GISS-E2-1-H	r1i1p1f1			144 x 90	Kelly et al. 2020
GISS-E2-1-H	r1i1p1f2			144 x 90	Kelly et al. 2020
GISS-E2-1-H	r1i1p3f1			144 x 90	Kelly et al. 2020
GISS-E2-1-H	r1i1p5f1			144 x 90	Kelly et al. 2020
GISS-E2-2-H	r1i1p1f1			144 x 90	Kelly et al. 2020
IITM-ESM	r1i1p1f1	Indian Institute of Tropical Meteorology	India	44 x 192	Krishnan et al. 2019
MIROC6	r1i1p1f1	Japan Agency for Marine-Earth Science and Technology, Atmosphere and Ocean Research Institute	Japan	256 x 128	Tatebe et al. 2019
MIROC-ES2L	r1i1000p1f2			128 x 64	Hajima et al. 2020
MIROC-ES2L	r1i1p1f2			128 x 64	Hajima et al. 2020
MRI MRI-ESM2-0	piControl			128 x 64	Yukimoto et al. 2023
MPI-ESM-1-2-HAM	r1i1p1f1	Max Planck Institute for Meteorology	Germany	192 x 96	Neubauer et al. 2019
TaiESM1	r1i1p1f1	Academia Sinica -Research Center for Environmental Changes	China (Republic of)	192 x 288	Lee et al. 2020

Table S2: Chronology statistics for five sites in northern Minnesota.

CFC	Detrending type	Start year	End year	N trees	N cores	Rbar	EPS
Picea mariana	100 year spline	1889	2019	15	28	0.47	0.941
Pinus resinosa	Negative exponential	1766	2019	15	29	0.57	0.963
MEF							
Larix laricina	100 year spline	1918	2019	15	30	0.72	0.982
Picea mariana	100 year spline	1878	2019	15	30	0.35	0.917
Pinus resinosa	100 year spline	1948	2019	15	30	0.89	0.995

Table S3: Classification scheme for northern forest types from USDA forest inventory data.

**Name**	**Scientific Name**	**Classification**
Jack Pine	*Pinus banksiana*	Upland conifer
Red Pine	*Pinus resinosa*	Upland conifer
Eastern hemlock	*Tsuga canadensis*	Upland conifer
Balsam fir	*Abies balsamea*	Upland conifer
Norway spruce	*Picea abies*	Upland conifer
Eastern white pine	*Pinus strobus*	Upland conifer
Black spruce	*Picea mariana*	Lowland conifer
Tamarack	*Larix laricina*	Lowland conifer
Northern white cedar	*Thuja occidentalis*	Lowland conifer
White ash	*Fraxinus americana*	Upland broadleaves
White oak	*Quercus alba*	Upland broadleaves
Red maple	*Acer rubrum*	Upland broadleaves
Aspen	*Populus tremuloides*	Upland broadleaves
Paper birch	*Betula papyrifera*	Upland broadleaves
Burr oak	*Quercus macrocarpa*	Oak savannah
Northern red oak	*Quercus rubra*	Oak savannah

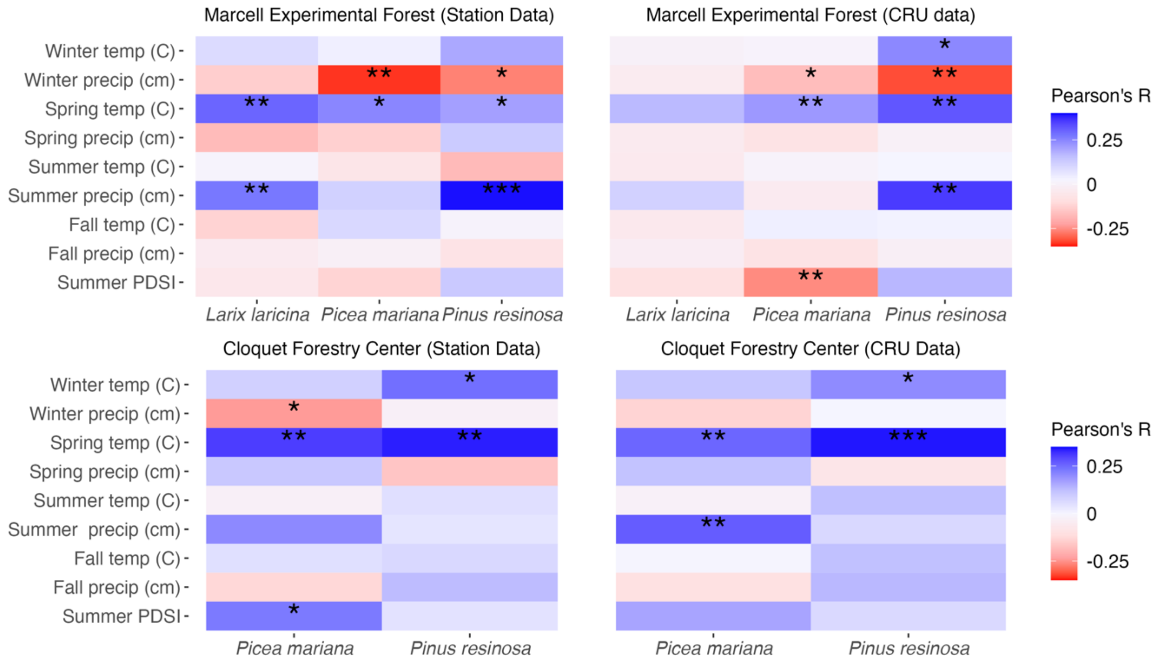

Fig. S1 *Climate-growth responses for tree species at two three northern Minnesota site locations. Shown are sensitivity to climate (both temperature and precipitation) year-round. I analyzed both station and CRU 4.06 data, as well as the CRU Summer Palmer Drought Severity Indices (PDSI). Asterisks indicate the output from subjecting the data to simple linear regression analysis:* * = $p < 0.05$, ** = $p < 0.01$, *** = $p < 0.001$.

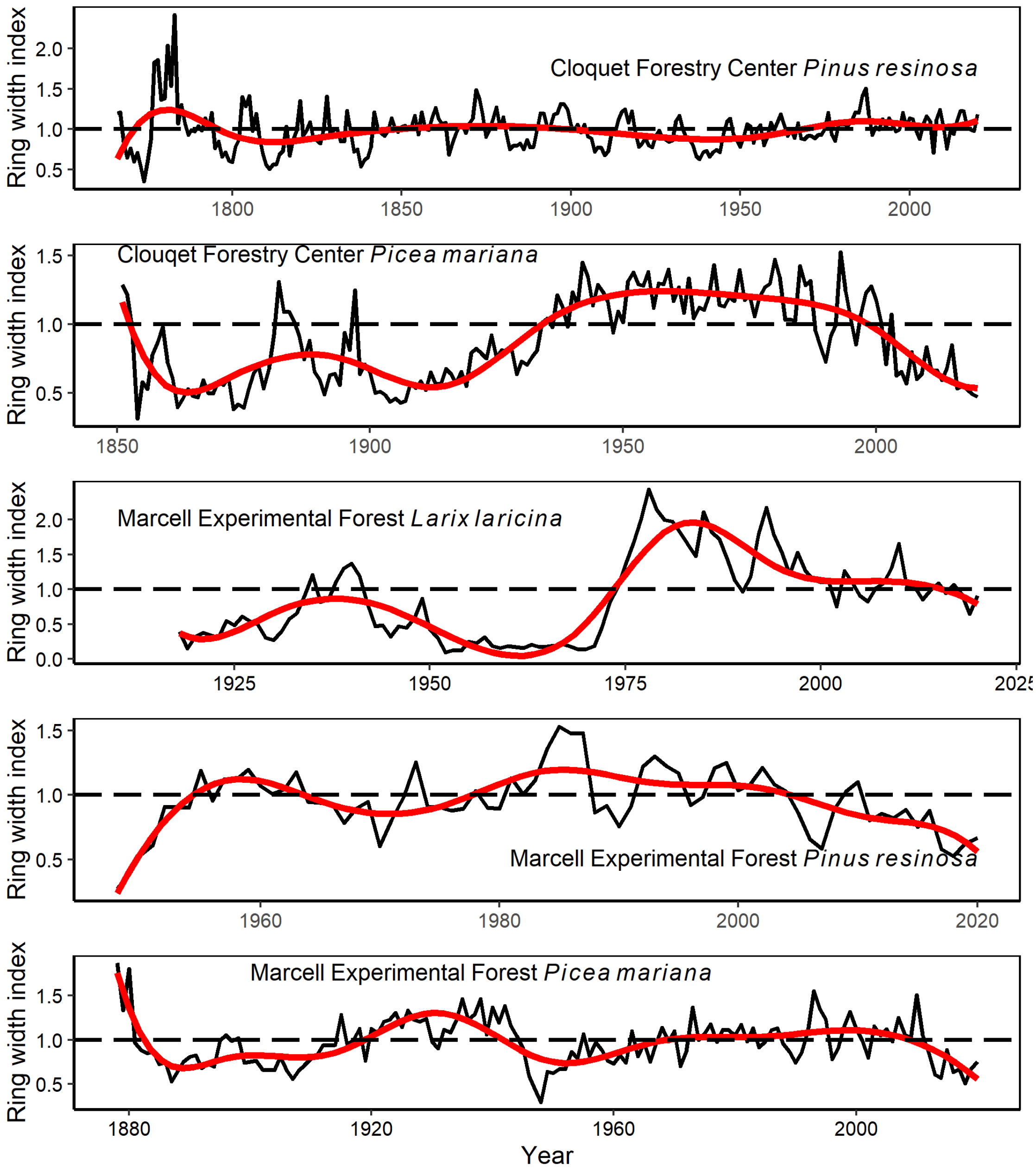

Fig. S2 *Chronologies of ring-width indices for all five tree-ring sites, detrended. The CFC Pinus resinosa site was detrended using a negative exponential curve, and the other three sites with 100 year cubic splines. The red line is a spline intended to illustrate the decadal-scale patterns in the data. Periodic growth releases are evident, particularly following the 1920s at the lowland Picea mariana site at Cloquet Forestry Center, and in the 1970s at the Larix laricina site at Marcell Experimental Forest.*

References

Cherchi A, Fogli PG, Lovato T, et al (2019) Global Mean Climate and Main Patterns of Variability in the CMCC-CM2 Coupled Model. Journal of Advances in Modeling Earth Systems 11:185–209. https://doi.org/10.1029/2018MS001369

Christian JR, Denman KL, Hayashida H, et al (2022) Ocean biogeochemistry in the Canadian Earth System Model version 5.0.3: CanESM5 and CanESM5-CanOE. Geoscientific Model Development 15:4393–4424. https://doi.org/10.5194/gmd-15-4393-2022
Danabasoglu G, Lamarque J-F, Bacmeister J, et al (2020) The Community Earth System Model Version 2 (CESM2). Journal of Advances in Modeling Earth Systems 12:e2019MS001916. https://doi.org/10.1029/2019MS001916
Danek C, Shi X, Stepanek C, et al (2020) AWI AWI-ESM1.1LR model output prepared for CMIP6 CMIP historical
Hajima T, Watanabe M, Yamamoto A, et al (2020) Development of the MIROC-ES2L Earth system model and the evaluation of biogeochemical processes and feedbacks. Geoscientific Model Development 13:2197–2244. https://doi.org/10.5194/gmd-13-2197-2020
Kelley M, Schmidt GA, Nazarenko LS, et al (2020) GISS-E2.1: Configurations and Climatology. Journal of Advances in Modeling Earth Systems 12:e2019MS002025. https://doi.org/10.1029/2019MS002025
Krishnan R, Swapna P, Vellore R, et al (2019) The IITM Earth System Model (ESM): Development and Future Roadmap. In: Randall DA, Srinivasan J, Nanjundiah RS, Mukhopadhyay P (eds) Current Trends in the Representation of Physical Processes in Weather and Climate Models. Springer, Singapore, pp 183–195
Lee W-L, Wang Y-C, Shiu C-J, et al (2020) Taiwan Earth System Model Version 1: description and evaluation of mean state. Geoscientific Model Development 13:3887–3904. https://doi.org/10.5194/gmd-13-3887-2020
Li L, Yu Y, Tang Y, et al (2020) The Flexible Global Ocean-Atmosphere-Land System Model Grid-Point Version 3 (FGOALS-g3): Description and Evaluation. Journal of Advances in Modeling Earth Systems 12:e2019MS002012. https://doi.org/10.1029/2019MS002012
Mackallah C, Chamberlain MA, Law RM, et al (2022) ACCESS datasets for CMIP6: methodology and idealised experiments. JSHESS 72:93–116. https://doi.org/10.1071/ES21031
Neubauer D, Ferrachat S, Siegenthaler-Le Drian C, et al (2019) HAMMOZ-Consortium MPI-ESM1.2-HAM model output prepared for CMIP6 CMIP historical
Rong X (2019) IPCC DDC: CAMS CAMS_CSM1.0 model output prepared for CMIP6 CMIP
Semmler T, Danilov S, Gierz P, et al (2020) Simulations for CMIP6 With the AWI Climate Model AWI-CM-1-1. Journal of Advances in Modeling Earth Systems 12:e2019MS002009. https://doi.org/10.1029/2019MS002009
Song Z, Qiao F, Bao Y, et al (2019) FIO-QLNM FIO-ESM2.0 model output prepared for CMIP6 CMIP historical
Swart NC, Cole JNS, Kharin VV, et al (2019) The Canadian Earth System Model version 5 (CanESM5.0.3). Geoscientific Model Development 12:4823–4873. https://doi.org/10.5194/gmd-12-4823-2019
Tatebe H, Ogura T, Nitta T, et al (2019) Description and basic evaluation of simulated mean state, internal variability, and climate sensitivity in MIROC6. Geoscientific Model Development 12:2727–2765. https://doi.org/10.5194/gmd-12-2727-2019
Wu T, Lu Y, Fang Y, et al (2019) The Beijing Climate Center Climate System Model (BCC-CSM): the main progress from CMIP5 to CMIP6. Geoscientific Model Development 12:1573–1600. https://doi.org/10.5194/gmd-12-1573-2019

Wu T, Zhang F, Zhang J, et al (2020) Beijing Climate Center Earth System Model version 1 (BCC-ESM1): model description and evaluation of aerosol simulations. Geoscientific Model Development 13:977–1005. https://doi.org/10.5194/gmd-13-977-2020

Yukimoto S, Koshiro T, Kawai H, et al (2019) IPCC DDC: MRI MRI-ESM2.0 model output prepared for CMIP6 CMIP piControl
